# Increase in serum prolactin levels as a potential biomarker in Crohn’s disease: a prospective cohort study

**DOI:** 10.3389/fmed.2026.1737904

**Published:** 2026-01-19

**Authors:** Mengjie Lu, Minmin Xu, Xinyi Tang, Lichao Qiao, Hongjin Chen, Bolin Yang

**Affiliations:** 1 Department of Colorectal Surgery / IBD Center, The Affiliated Hospital of Nanjing University of Chinese Medicine, Nanjing, China; 2 First Clinical Medical College, Nanjing University of Chinese Medicine, Nanjing, China

**Keywords:** C-reactive protein, Crohn’s disease, fecal calprotectin, inflammation, prolactin

## Abstract

**Background:**

Crohn’s disease (CD) management lacks biomarkers that precisely reflect immune activity. Prolactin (PRL) has immunomodulatory functions, but its role in CD is unclear.

**Aim:**

To investigate serum PRL changes in CD patients and evaluate its potential as a biomarker for disease activity and treatment response.

**Methods:**

In a prospective cohort study, serum PRL levels were analyzed in 185 CD patients and 58 healthy controls, and correlated with clinical markers (CRP, FC).

**Results:**

Serum PRL levels were significantly higher in CD patients versus controls (p<0.001). Higher baseline PRL levels correlated with a greater reduction in CRP after treatment. The study population had no previous reproductive history.

**Conclusion:**

Elevated serum PRL may serve as a supplementary biomarker reflecting immune dysregulation and disease activity in CD, particularly in this cohort enriched with severe, perianal-dominant disease. Its role in evaluating therapeutic effectiveness warrants further investigation.

## Introduction

Crohn’s disease (CD) is a chronic, relapsing inflammatory disorder of the gastrointestinal tract characterized by complex interactions among genetic susceptibility, immune dysregulation, and gut microbiota imbalance (1, 2). The disease involves aberrant activation of both innate and adaptive immune responses, leading to persistent mucosal inflammation and progressive tissue damage (3, 4). Despite significant advances in biological therapy, the unpredictable course and high relapse rate of CD continue to pose major challenges to long-term disease management (5, 6). Clinical evaluation often relies on biomarkers such as white blood cell (WBC) count, C-reactive protein (CRP) and fecal calprotectin (FC); however, these indicators reflect inflammation only indirectly and vary considerably among patients (7). The identification of new biomarkers that better mirror immune activity could improve disease monitoring and therapeutic precision (8).

Prolactin (PRL), a polypeptide hormone primarily secreted by the anterior pituitary, has emerged as a key immunomodulatory molecule beyond its classical endocrine role (9). PRL receptors are expressed on T and B lymphocytes, macrophages, and dendritic cells, enabling PRL to influence multiple stages of immune activation (9, 10). These effects are mediated through intricate intracellular signaling pathways downstream of the prolactin receptor (11). Previous studies have shown that PRL enhances IL-2 receptor expression, promotes T-cell proliferation, and modulates the Th1/Th2 cytokine balance (12). Interestingly, PRL exhibits dual immunological behavior—it can potentiate pro-inflammatory responses in autoimmune diseases such as systemic lupus erythematosus and rheumatoid arthritis, yet may also contribute to immune regulation and tissue repair under certain conditions (9, 12, 13). These findings suggest that PRL serves as a context-dependent mediator in immune homeostasis.

Although hyperprolactinemia has been documented in several autoimmune disorders, its role in Crohn’s disease remains poorly defined (12, 13). Existing studies have primarily focused on growth and pubertal development in adolescent CD patients, with limited data available for adults (14, 15). Moreover, no prospective studies have systematically examined the relationship between serum prolactin levels, inflammatory biomarkers, and therapeutic outcomes in adult CD cohorts. The absence of such evidence has hindered understanding of whether prolactin reflects immune activation, compensatory regulation, or treatment response in the inflammatory process of CD.

Therefore, this study aimed to evaluate the expression pattern of prolactin in adult patients with Crohn’s disease and to investigate its association with disease activity and post-treatment inflammatory improvement through a prospective cohort design.

## Materials and methods

### Patients

This prospective single-center observational study was conducted at the Jiangsu Province Hospital of Chinese Medicine (Nanjing, China) from May 2017 to February 2022.

A total of 185 patients with a confirmed diagnosis of Crohn’s disease (CD) according to World Health Organization criteria (WHO) were enrolled in the experimental cohort. All CD patients were aged between 16 and 40 years for females, or 16 and 45 years for males, and were diagnosed and managed by the specialized Inflammatory Bowel Disease Center of our hospital. For baseline comparison, 58 non-CD individuals were recruited from the Department of Colorectal Surgery during the same period to match the demographic profile. These controls underwent routine health check-ups or were hospitalized for benign, non-inflammatory anorectal conditions (e.g., hemorrhoids, anal fissures) and had no history or evidence of inflammatory bowel disease or other chronic gastrointestinal inflammatory disorders.

Inclusion criteria for all participants were provision of written informed consent and age as specified above. The exclusion criteria (applied to both groups) were: (1) Pregnancy, lactation, or any known condition that could substantially affect sex hormone levels (e.g., endocrine disorders such as pituitary adenoma, hypothyroidism, or Cushing’s syndrome). (2) History of severe cardiac, hepatic, renal dysfunction, or major cardiovascular disease prior to enrollment. (3) Use of medications known to significantly influence serum prolactin (PRL) levels within 3 months before enrollment, including dopamine receptor antagonists (e.g., antipsychotics, metoclopramide), certain antidepressants (e.g., SSRIs, tricyclic antidepressants), opioids, and oral contraceptives.

Additionally, for CD patients, we specifically verified that no factors existed (as listed above) that could cause significant fluctuations in sex hormones prior to the diagnosis of CD, ensuring the observed hormonal changes were more likely associated with the disease itself. Participants who voluntarily withdrew from the study during follow-up were also excluded from the final analysis.

The study protocol was approved by the Ethics Committee of Jiangsu Province Hospital of Traditional Chinese Medicine (approval no. 2019NL-063-030), and all procedures were conducted in accordance with the Declaration of Helsinki.

### Data collection

The general data from patients in the experimental and control groups, including age, sex, and reproductive history, were collected. Additional information regarding the patients in the experimental group was collected from the Electronic Medical Records System of the Jiangsu Province Hospital of Chinese Medicine, which included the Montreal Classification, CRP, ESR, FC, Perianal Disease Activity Index (PDAI), Crohn’s Disease Activity Index (CDAI), body mass index (BMI), serum albumin, hemoglobin, folate, surgical history, and history of biological agents.

Blood samples were collected in the early morning (8:00–10:00 a.m.), and for female participants, on the third day of menstruation. Serum prolactin (PRL) levels were determined using a chemiluminescence immunoassay (Access Prolactin assay, Beckman Coulter) on an Access/UniCel DxI series system. This assay is a two-site immunoenzymatic (“sandwich”) assay employing paramagnetic particles coated with a mouse monoclonal anti-PRL antibody and a goat polyclonal anti-PRL antibody conjugated to alkaline phosphatase. It primarily detects monomeric prolactin. According to the manufacturer’s insert, the established reference range for premenopausal women (<50 years) is 3.34–26.72 ng/mL. Considering that the female participants in our study were relatively young (aged 16–40 years), the sex-specific upper reference limits validated and used by our institutional clinical laboratory for this population were applied: >13.1 ng/mL for men and >25 ng/mL for women. PRL levels exceeding these limits were classified as elevated.

Among all enrolled Crohn’s disease patients, those rehospitalized within 14–30 weeks for treatment or diagnostic purposes were included in follow-up assessments. At each visit, disease activity, treatment regimen, and laboratory indices (PRL, CRP, Hb, albumin, FC) were reassessed. Dynamic changes in PRL were analyzed alongside inflammatory indicators and clinical scores (CDAI and PDAI) to explore whether PRL could serve as a potential predictor of disease activity and therapeutic efficacy.

All patients included in the experimental group were diagnosed with CD using a specialized medical structure and met the World Health Organization criteria (16) for the diagnosis of CD. Patients with CD were classified based on the universal classification criteria for the Montreal typology (17) and the PDAI (18, 19).

To ensure that observed prolactin (PRL) alterations were more specifically associated with Crohn’s disease rather than confounded by other common systemic conditions, we proactively collected and scrutinized additional laboratory parameters and medical history. Renal function was assessed by serum creatinine, and hematologic diseases were screened via white blood cell (20, 21). Furthermore, a detailed review of medical history, imaging reports (where available), and clinical presentations was conducted to exclude participants with known or suspected pituitary lesions, such as prolactinomas, in accordance with current diagnostic consensus (22). This comprehensive approach aimed to minimize the influence of renal, hematologic, and primary pituitary disorders on serum PRL levels. Given that endocrine dysfunction, including abnormalities in the thyroid and other axes, has been documented even in pediatric CD populations (14), our stringent exclusion of known endocrine disorders was crucial to isolate the relationship between PRL and intestinal inflammation.

However, it is important to acknowledge the study’s limitations in this regard. Due to the observational nature of this study and its primary focus on Crohn’s disease activity, systematic pituitary imaging (e.g., MRI) and dynamic testing of other endocrine axes (such as thyroid-stimulating hormone, TSH) were not part of the standardized protocol for all participants. While our meticulous history review aimed to exclude clinically apparent endocrine disorders, we recognize that the absence of these objective measures means subclinical pituitary or other endocrine pathologies could not be definitively ruled out as potential confounders for PRL levels. This limitation is considered in the interpretation of our findings.

### Statistical analysis

All statistical analyses and graphical representations were performed using SPSS (version 25.0; SPSS, Chicago, IL, USA) and GraphPad Prism 9 (GraphPad Software, San Diego, USA).

Categorical variables were expressed as numbers (percentages) and continuous variables as mean ± SD or median (IQR). Chi-square tests and *t*-tests or Mann–Whitney U tests were used for group comparisons. Binary logistic regression was applied to identify predictors of PRL elevation, with results presented as odds ratios (ORs) and 95% confidence intervals (CIs).

All analyses were performed using SPSS 25.0 and GraphPad Prism 9.0, with *p* < 0.05 considered statistically significant. The optimal probability cutoff for logistic regression models was determined by maximizing the Youden index.

Prolactin concentrations are reported to one decimal place in accordance with the analytical precision of the immunoassay.

## Results

### Baseline characteristics

**Figure 1:**
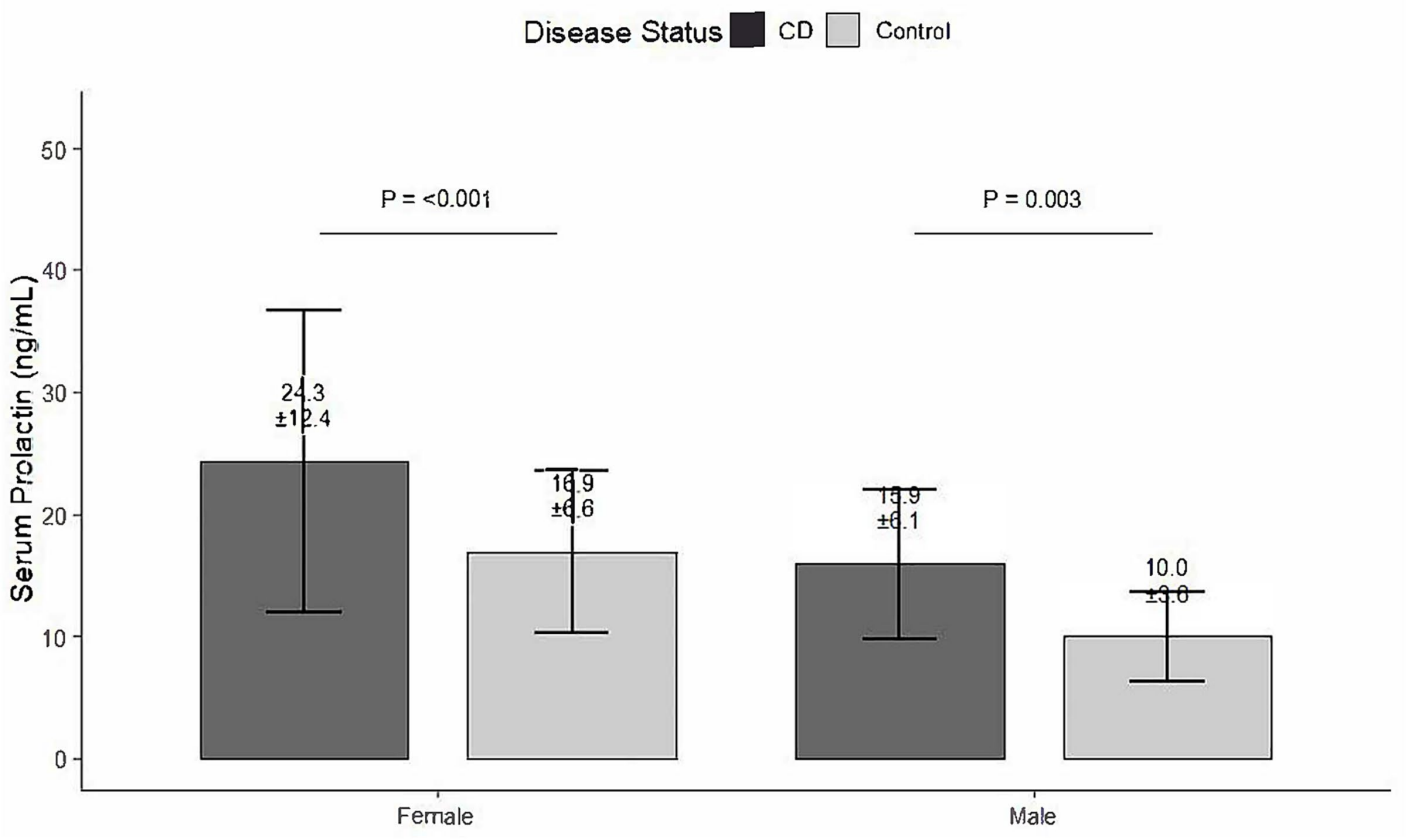
Serum prolactin levels in Crohn’s disease patients and healthy controls, stratified by sex. Bar graph showing serum prolactin concentrations (mean ± SD) in patients with Crohn’s disease (CD) and healthy controls, presented separately for males and females. Significant differences between CD patients and controls within the same sex group are indicated (*p* values shown above brackets; independent samples *t*-test). CD, Crohn’s disease.

A total of 185 patients with Crohn’s disease (134 males [72%] and 51 females [28%]) were enrolled in the experimental group, while 58 non-CD individuals (39 males [67%] and 19 females [33%]) served as controls. The median age was 26.6 years (IQR 16–44) in the CD group and 29.8 years (IQR 16–45) in the control group. Regarding reproductive history, 129 CD patients (69.7%) and 16 controls (27.6%) were nulliparous, showing a significant difference between groups (*p* < 0.001). The median serum prolactin (PRL) level was 18.3 ng/mL (IQR 5.1–99.9) in CD patients and 12.3 ng/mL (IQR 4.6–33.8) in controls, also significantly higher in the CD group (*p* < 0.001). A graphical comparison of serum prolactin levels between groups and subgroups was presented in Figure 1. Additionally, renal function and routine hematological parameters were comparable between the two groups: serum creatinine levels (CD: 70.21 ± 13.54 μmol/L, control: 71.48 ± 12.52 μmol/L, *p* = 0.570) and white blood cell counts (CD: 6.45 ± 1.77 × 10⁹/L, control: 6.06 ± 1.69 × 10^9^/L, *p* = 0.182) showed no significant difference. The vast majority of participants had values within the normal reference range for both parameters (normal creatinine: 100% in both groups; normal WBC: 96.1% in CD group, 97.3% in control group). Overall, 112 participants (46%) in the total cohort had elevated PRL levels, whereas 131 (54%) showed normal PRL concentrations (Table 1). Notably, approximately 95% of the CD patients in this cohort had a history of perianal surgery, which is much higher than reported in typical CD populations. This indicates that our study population is heavily enriched for patients with severe, perianal-dominant Crohn’s disease, and thus the findings should be interpreted with caution regarding generalizability to broader CD populations.

**Table 1:** Patient baseline characteristics.

Variable	CD (*n* = 185)	Controls (*n* = 58)	*p*
Age (years)	26.60 ± 6.67	29.84 ± 5.47	0.001
Female age (years)	27.84 ± 6.85	29.63 ± 4.02	0.289
Gender			0.273
Male *n* (%)	134 (72.4)	39 (67.2%)	
Female *n* (%)	51 (27.6)	19 (32.8%)	
Reproductive history			<0.001
Yes *n* (%)	56 (30.3%)	42 (72.4%)	
No *n* (%)	129 (69.7%)	16 (27.6%)	
Prolactin (ng/ml)	18.3 ± 9.1	12.3 ± 5.8	<0.001
Creatinine (μmol/L)			
Value, mean ± SD	70.21 ± 13.54 (*n* = 51)	71.48 ± 12.52 (*n* = 111)	0.570
Normal, *n* (%)	51 (100.0)	111 (100.0)	
Abnormal, *n* (%)	0 (0.0)	0 (0.0)	
White blood cell (×10⁹/L)			
Value, mean ± SD	6.45 ± 1.77 (*n* = 51)	6.06 ± 1.69 (*n* = 111)	0.182
Normal, *n* (%)	49 (96.1)	108 (97.3)	
Abnormal, *n* (%)	2 (3.9)	3 (2.7)	

Notably, the control group had a significantly higher proportion of participants with a history of childbirth compared to the CD group (72.4% vs. 30.3%, *p* < 0.001). This difference reflects a disparity in reproductive stage that may influence PRL levels and is consistent with the well-documented epidemiology of Crohn’s disease, where patients—particularly those with active, severe, or surgically treated disease—often have lower fertility rates and fewer pregnancies compared to the general population (23, 24).

### Effect of baseline prolactin status on treatment response at follow-up

**Figure 2:**
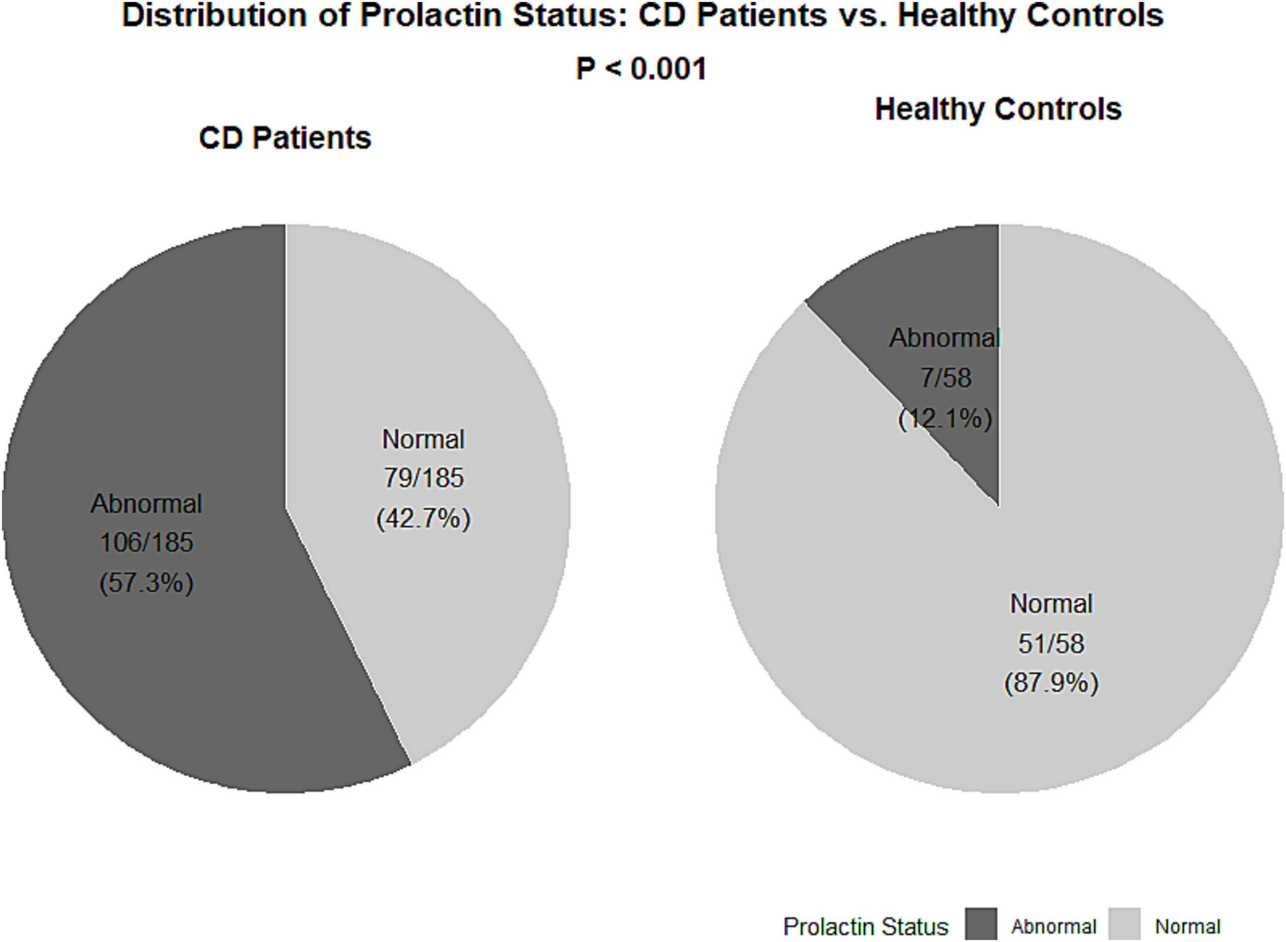
Distribution of prolactin status in Crohn’s disease patients and healthy controls. Two pie charts show the proportion of participants with abnormal or normal serum prolactin levels within the Crohn’s disease (CD) patient group (left) and the healthy control group (right). The *p* value (derived from Chi-square test) above the charts indicates the significant difference in the prevalence of abnormal prolactin between the two groups. CD, Crohn’s disease.

Prolactin normalization was defined as a reduction from an elevated level at baseline to within the normal reference range at the follow-up assessment. Patients with CD showed a significant increase in PRL levels (*T* = 5.91, *p* < 0.001) and a significant decrease in the reproductive history (*X*^2^ = 32.54, *p* < 0.001). No significant differences were observed regarding sex (*X*^2^ = 0.57, *p* = 0.51) or female age (*p* = 0.289). After dividing all patients into two groups according to the PRL level, a significant difference in sex, age (*T* = −3.94, *p* < 0.001), and CD (*X*^2^ = 36.31, *p* < 0.001) was observed in the group with abnormal PRL levels. PRL values were assigned as normal or abnormal depending on the reference PRL levels for men and women. A total of 106 patients with CD (57.2%) in the experimental group had abnormal PRL levels, while only 7 patients (12%) in the control group had abnormal PRL levels (Figures 1, 2).

### Factors associated with PRL in patients with Crohn’s disease

Baseline data for all 185 patients with CD were recorded in quantitative or qualitative detail, including sex, age, Montreal staging, indicators of specific inflammation, and nutritional status, as well as previous modalities of treatment received (Table 2) for inclusion in correlation and single multifactorial analyses.

**Table 2:** Baseline data of Crohn’s disease patients divided by prolactin level.

Baseline characteristics	Total *n* = 185		Normal *n* = 79	Abnormal *n* = 106
Age (years)		27.37 (16–44)	28.39 (16–41)	25.26 (16–44)
Gender (male)	*n* (%)	134 (72%)	49 (62.0%)	85 (80.2%)
Montreal classification				
A1	*n* (%)	8 (4%)	1 (1.3%)	7 (6.6%)
A2	*n* (%)	176 (95%)	78 (98.7%)	98 (92.5%)
A3	*n* (%)	1 (1%)	0 (0%)	1 (0.9%)
L1	*n* (%)	60 (32%)	25 (31.6%)	35 (33%)
L2	*n* (%)	35 (18%)	18 (22.8%)	17 (16%)
L3	*n* (%)	89 (48%)	36 (45.6%)	53 (50%)
L4	*n* (%)	1 (1%)	0 (0%)	1 (9%)
B1	*n* (%)	127 (68%)	49 (62%)	79 (74.5%)
B2	*n* (%)	51 (27%)	28 (35.4%)	23 (21.7%)
B3	*n* (%)	4 (2%)	1 (1.3%)	3 (2.8%)
B2 + B3	*n* (%)	2 (1%)	1 (1.3%)	1 (0.9%)
P1	*n* (%)	174 (94%)	73 (92.4%)	101 (95.3%)
Abdominal operation	*n* (%)	15 (8%)	8 (10.1%)	7 (6.6%)
Perianal operation	*n* (%)	176 (95.1%)	74 (93.7%)	102 (92.6%)
PRL (ng/ml)	Mean	18.3 (5.1–99.9)	12.6(5–24)	22.5 (13–67)
CRP (mg/L)	Mean	21.93 (1–222)	23.96 (1–222)	20.43 (1–113)
ESR (mm/h)	Mean	30 (2–118)	29.76 (2–118)	29.67 (2–100)
FC (μg/g feces)	Mean	653.4 (5.5–4,049)	793.47 (12–4,049)	562.62 (6–1,525)
CDAI	Mean	92.53 (0–341)	96.80 (6–341)	89.35 (0–271)
PDAI	Mean	4 (0–14)	3.80 (0–14)	4.06 (0–11)
Folic acid (μg)	Mean	7.41 (1–20)	8.41 (1–20)	6.71 (1–20)
Hb (g/dL)	Mean	124 (66–173)	121.58 (71–157)	125.54 (66–173)
Alb (g/dL)	Mean	38.89 (24.1–65.94)	38.43 (25–62)	39.23 (24–66)
BMI (kg/m^2^)	Mean	20.32 (13–29.7)	20.66 (16–28)	20.07 (13–30)

CD, Crohn’s disease; PRL, prolactin; CRP, C-reactive protein; ESR, erythrocyte sedimentation rate; FC, fecal calprotectin; CDAI, Crohn’s Disease Activity Index; PDAI, Perianal Disease Activity Index; Hb, hemoglobin; Alb, albumin; BMI, body mass index. Montreal classification: A1 (<17 years), A2 (17–40 years), A3 (>40 years); L1 (ileal), L2 (colonic), L3 (ileocolonic), L4 (upper GI involvement); B1 (non-stricturing, non-penetrating), B2 (stricturing), B3 (penetrating); B2 + B3 indicates mixed-type behavior with both stricturing and penetrating features. P1 denotes the presence of perianal disease. Data are presented as median (range) or *n* (%).

**Figure 3:**
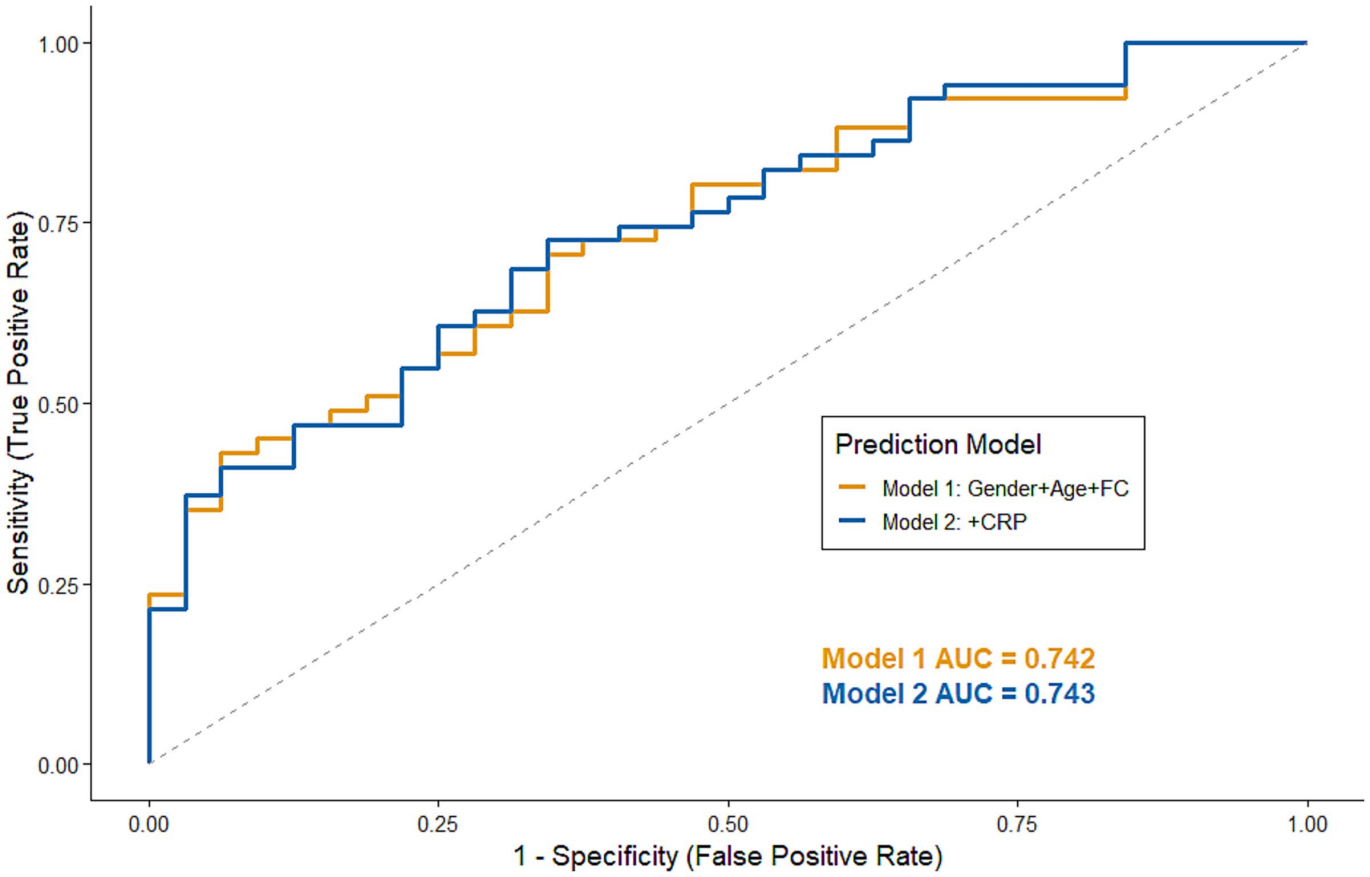
ROC curve of logistic regression at baseline. Receiver operating characteristic (ROC) curves for predicting abnormal prolactin levels. Based on 83 complete cases, the curves illustrate the discriminatory performance of two prediction models: Model 1 (Gender + Age + FC, AUC = 0.742) and Model 2 (Model 1 + CRP, AUC = 0.743). The close proximity of the two curves suggests that the addition of CRP provides limited improvement in predictive performance.

Table 3 shows the clinical characteristics of the 185 patients with CD based on normal or non-normal PRL levels. Significant differences were found in age (*p* < 0.001), sex (*p* = 0.016), reproductive history (*p* = 0.001), FC (*p* = 0.017), and folic acid (*p* = 0.035) levels between patients with CD with normal and abnormal PRL levels. However, the two groups showed similar distributions of hematocrit, CRP, CDAI, PDAI, hemoglobin, albumin, BMI, age at diagnosis, Montreal staging, treatment options for biological agents, and surgery (all *p* > 0.05). Possible clinical characteristics (*p* < 0.20) were incorporated into a logistic multifactorial analysis. The analysis revealed that older age (OR = 0.898, *p* = 0.011) and increased FC levels (OR = 0.999, *p* = 0.012) were significantly associated with a lower likelihood of having abnormal PRL levels. In the ROC validation curve after logistic prediction based on the above clinical characteristics, the area under the curve was 0.742 (Figure 3).

**Table 3:** Univariate and multivariate analyses of factors associated with abnormal prolactin levels in Crohn’s disease patients at baseline.

Variables	Univariate analysis		Multivariate analysis	
	OR (95% CI)	*p* value	OR (95% CI)	*p* value
Age (years)	0.932 (0.886–0.962)	<0.001	0.896 (0.826–0.971)	0.011
Gender (male)	2.031 (1.141–3.615)	0.016	2.386 (0.998–5.702)	0.05
Reproductive history	0.413 (0.242–0.702)	0.001	2.047 (0.699–5.989)	0.191
ESR (mm/h)	1.000 (0.989–1.011)	0.983		
FC (μg/g feces)	0.999 (0.999–1.000)	0.017	0.999 (0.998–1.000)	0.011
CRP (mg/L)	0.996 (0.986–1.006)	0.435		
CDAI	0.998 (0.993–1.003)	0.402		
PDAI	1.031 (0.933–1.139)	0.553		
Folic acid (μg)	0.936 (0.981–0.995)	0.035	0.942 (0.872–1.017)	0.125
Hb (g/dL)	1.009 (0.995–1.023)	0.205		
Alb (g/dL)	1.024 (0.973–1.077)	0.366		
BMI (kg/m^2^)	0.946 (0.865–1.035)	0.225		
Lesion	1.064 (0.768–1.473)	0.710		
Behavior	0.709 (0.428–1.177)	0.183	1.067 (0.563–2.023)	0.842
Perianal	1.660 (0.488–5.649)	0.417		
Biologicals	0.904 (0.504–1.623)	0.736		
Abdominal operation	1.007 (0.353–2.870)	0.989		
Perianal operation	1.090 (0.286–4.161)	0.900		

### Effects of prolactin on treatment outcome at the follow-up

Information on 99 patients (male, 71; female, 28) was collected at the 14–30 weeks follow-up. Sixty-seven patients comprised the abnormal PRL group, whereas the other 32 patients had normal PRL levels. During the follow-up period, 6 patients underwent abdominal surgery (6.1%), 96 patients underwent perianal surgery (97%), and 95 patients were in the course of therapy (95.9%) during their hospitalization.

**Figure 4:**
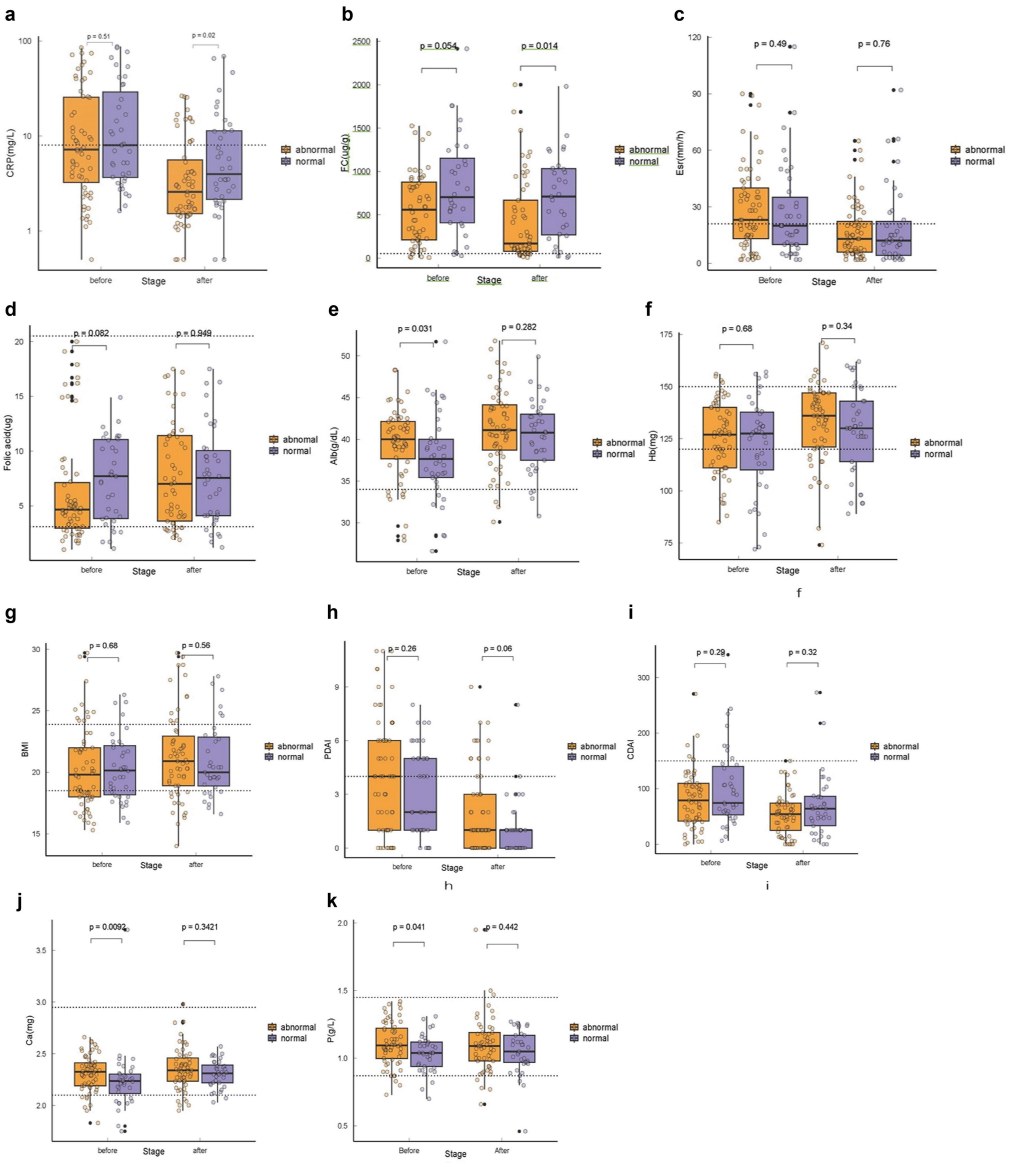
Comparison of inflammatory and nutritional markers after treatment, stratified by baseline prolactin status. Box plots show the distribution of **(A)** C-reactive protein (CRP), **(B)** fecal calprotectin (FC), **(C)** erythrocyte sedimentation rate (ESR), **(D)** folic acid, **(E)** albumin (Alb), **(F)** hemoglobin (Hb), **(G)** body mass index (BMI), **(H)** Perianal Disease Activity Index (PDAI), and **(I)** Crohn’s Disease Activity Index (CDAI) in Crohn’s disease patients at the follow-up visit (14–30 weeks after treatment initiation). Patients are grouped according to their serum prolactin (PRL) level at baseline: normal PRL (≤13.13 ng/mL for men, ≤25 ng/mL for women) vs. elevated PRL. The dashed lines indicate the upper or lower limits of normal reference ranges for each laboratory parameter. Data are presented as mean ± SD or median (IQR), as appropriate. *p < 0.05, **p < 0.01, ***p < 0.001 for comparisons between groups (Mann–Whitney U test or independent *t*-test).

Serum prolactin and other biomarkers were measured at baseline and reassessed at a single follow-up visit within 14–30 weeks after treatment initiation. After 14 to 30 weeks of treatment cycles, patients in the abnormal PRL group had significantly lower CRP levels than those in the normal PRL group (*p* = 0.002). Furthermore, FC levels were significantly lower in the abnormal PRL group (*p* = 0.014), whereas other markers of inflammation and indicators of nutritional status did not show any significant differences between the two groups after treatment (all *p* > 0.05) (Figure 4).

**Figure 5:**
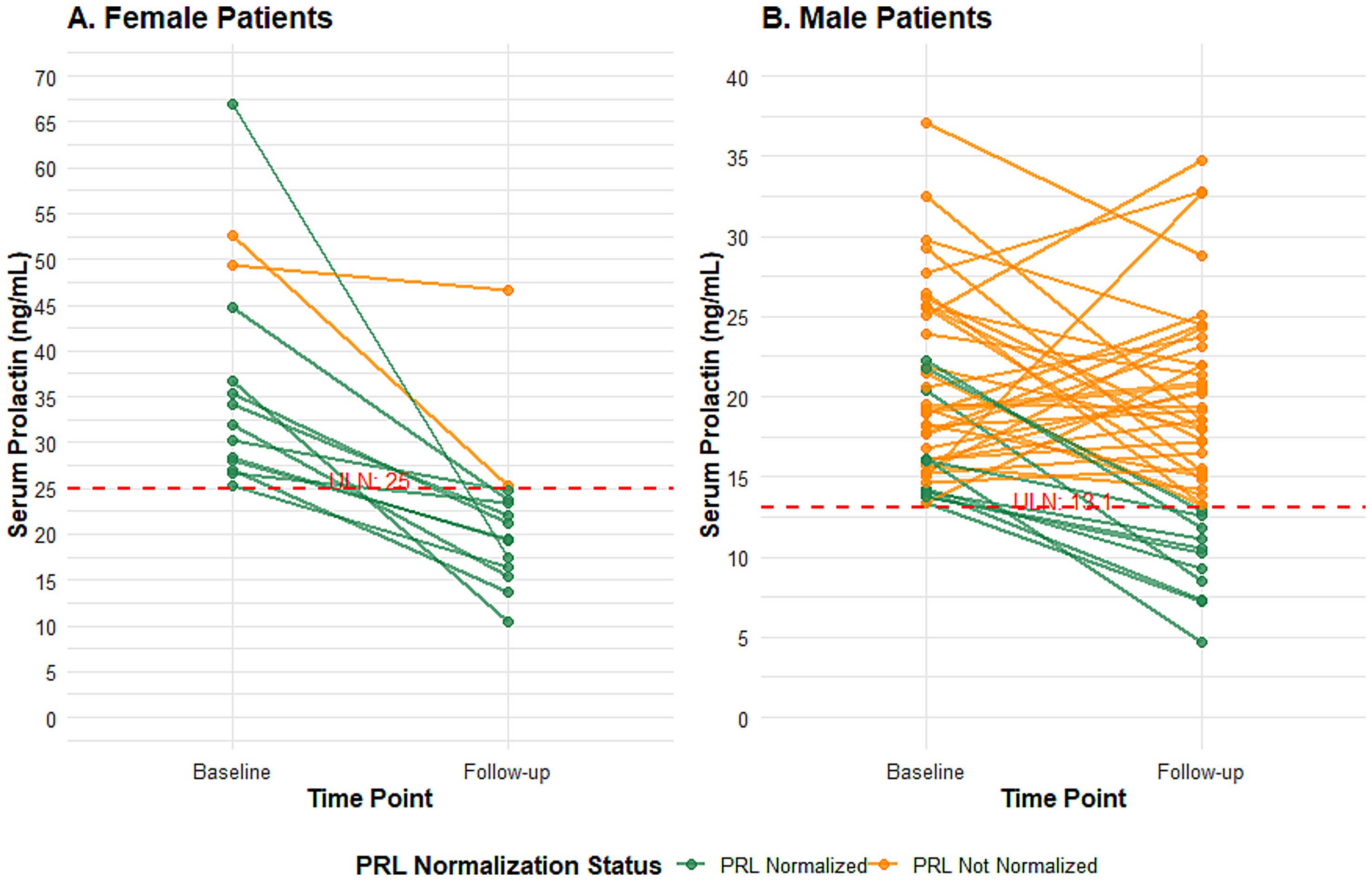
Longitudinal changes in serum prolactin levels from baseline to follow-up after treatment initiation, stratified by sex and normalization status. **(A)** Female patients (*n* = 14). **(B)** Male patients (*n* = 47). Individual lines connect serum prolactin (PRL) levels measured at baseline and at a single follow-up visit (14–30 weeks after treatment initiation). Patients are grouped based on their PRL status at follow-up: those whose elevated baseline PRL returned to within the sex-specific normal range (PRL normalized, blue lines) and those whose PRL remained elevated (PRL not normalized, red lines). The dashed horizontal line in each panel indicates the upper limit of normal (ULN) for serum PRL (13.13 ng/mL for men, 25 ng/mL for women). The graph visually underscores the pronounced sex difference in the rate and pattern of PRL normalization observed during therapy.

To visualize the individual response patterns, longitudinal trajectories of serum PRL levels are presented in Figure 5. Among the 61 patients analyzed, 14 were female and 48 were male. A striking disparity was observed: 12 of 14 female patients (85.7%) achieved PRL normalization at follow-up, and all of them showed a clear downward trend. In contrast, only 13 of 47 male patients (27.6%) achieved normalization. Notably, the male cohort as a whole did not demonstrate a consistent pattern of decline, with many trajectories showing fluctuation or persistent elevation above the gender-specific upper limit of normal (ULN). This graphical analysis underscores a pronounced gender difference in both the rate of PRL normalization and the pattern of its change during therapy. The differential trajectories suggest that the administered treatments did not uniformly suppress PRL levels. Instead, PRL normalization appeared to be intrinsically linked to a more favorable anti-inflammatory response, which was markedly more prevalent in female patients (Figure 5).

**Figure 6:**
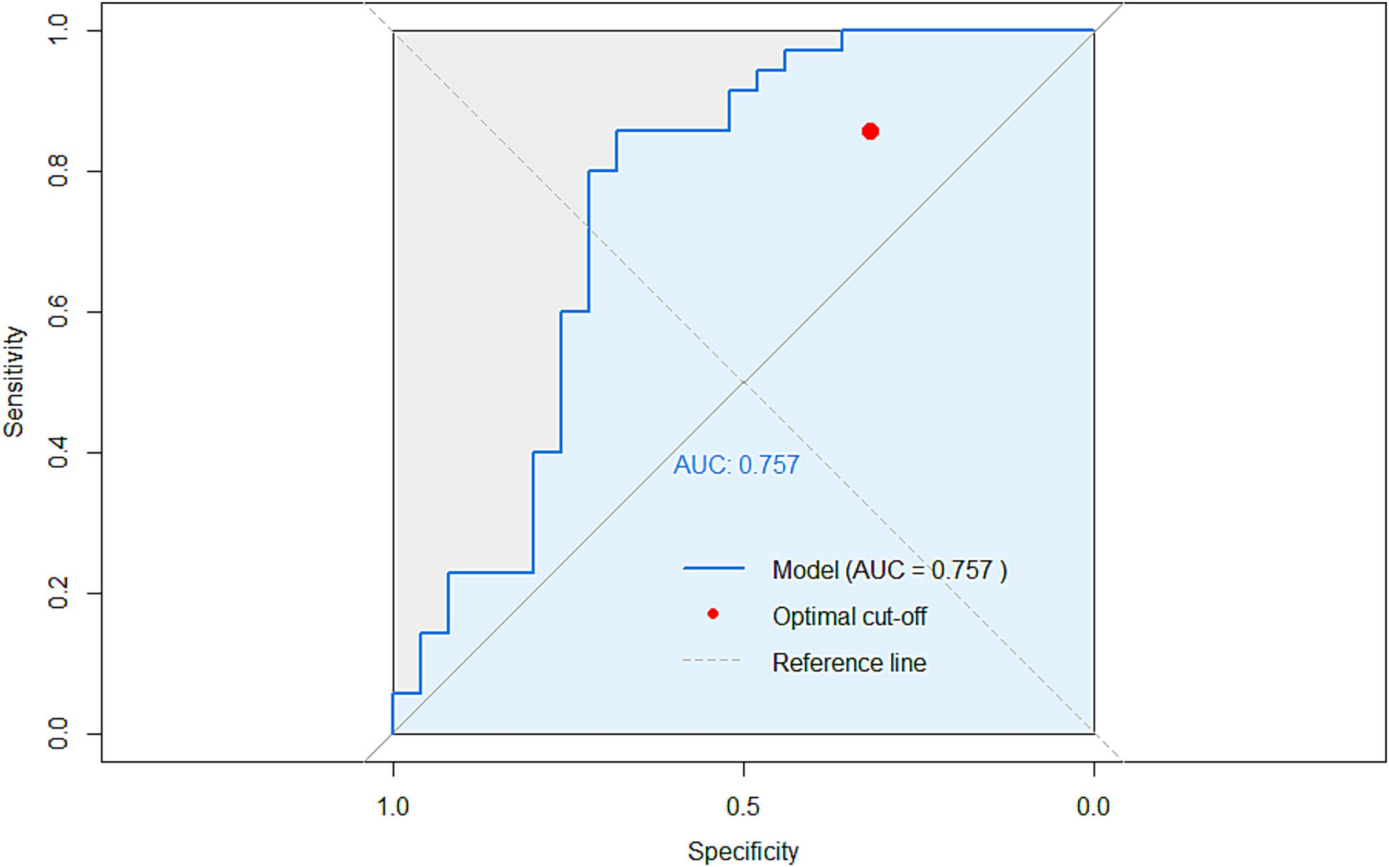
Receiver operating characteristic (ROC) curve for predicting prolactin normalization after treatment. The ROC curve illustrates the performance of a logistic regression model (incorporating follow-up C-reactive protein [CRP], hemoglobin [Hb], and gender) in discriminating patients whose elevated baseline serum prolactin (PRL) levels returned to the normal reference range after 14–30 weeks of therapy. The analysis included 60 patients (35 with abnormal PRL at baseline). The area under the curve (AUC) is 0.757. The red circle indicates the optimal probability cutoff point determined by the Youden index (threshold = 0.652), corresponding to a sensitivity of 85.7% and a specificity of 68.0%.

Focusing on the 61 patients with abnormal baseline PRL, PRL levels returned to normal after treatment in 25 patients (40.9%), while levels remained elevated in the remaining 36 patients (59.1%). Logistic regression analysis of the two groups revealed that high CRP levels (OR = 1.034, *p* = 0.44) and high Hb values (OR = 1.078, *p* = 0.002) were risk factors for abnormal PRL maintenance (Table 4). The area under the curve in the logistically predicted roc validation curve based on the follow-up data was 0.757 (Figure 6).

**Table 4:** Univariate and multivariate analyses at follow-up.

Variables	Univariate analysis		Multivariate analysis	
	OR (95% CI)	*p* value	OR (95% CI)	*p* value
Ca	1.310 (0.07–24.517)	0.857		
P	0.400 (0.016–9.893)	0.576		
ESR	1.008 (0.984–1.032)	0.515		
FC	1.000 (0.999–1.002)	0.802		
CRP	1.018 (0.991–1.046)	0.184	1.034 (1.001–1.068)	0.044
CDAI	0.997 (0.988–1.007)	0.608		
PDAI	1.030 (0.880–1.206)	0.708		
Folic acid	1.003 (0.902–1.115)	0.957		
Hb	1.039 (1.006–1.073)	0.019	1.078 (1.028–1.131)	0.002
Alb	0.935 (0.841–1.039)	0.21		
BMI	0.958 (0.822–1.117)	0.584		

## Discussion

The present prospective study demonstrated that prolactin (PRL) levels were significantly elevated in patients with Crohn’s disease compared with non-CD controls, and that abnormal PRL expression was associated with distinct inflammatory profiles and treatment responses. Furthermore, dynamic changes in PRL correlated with improvements in C-reactive protein (CRP) levels following therapy, suggesting that PRL may serve as a potential biomarker reflecting immune and inflammatory regulation in Crohn’s disease.

Previous studies have primarily focused on classical inflammatory markers such as CRP and fecal calprotectin (FC) for evaluating disease activity in CD (25, 26). However, these indicators reflect systemic or mucosal inflammation indirectly and are influenced by treatment regimens, disease location, and individual metabolic variability (7). Our findings highlight that PRL—a hormone traditionally linked to lactation—may also participate in intestinal immune homeostasis. Elevated PRL levels have been described in several autoimmune and inflammatory diseases, including systemic lupus erythematosus and rheumatoid arthritis, where PRL modulates T-cell proliferation, cytokine release, and antigen presentation (10, 27). The current results extend this understanding to Crohn’s disease, suggesting that PRL elevation may reflect activation of immunoregulatory pathways during chronic intestinal inflammation (15, 28). In our study, higher fecal calprotectin (FC) levels were observed to be associated with normal prolactin (PRL) levels, which may appear contradictory to the conventional understanding that elevated inflammation typically leads to abnormal PRL expression(OR = 0.999, *p* = 0.012). However, our data showed that both groups of patients, regardless of PRL status, had elevated FC levels, with the normal PRL group having a mean PRL level of 793.47 ± 89.94 ng/mL and the abnormal PRL group having a mean of 562.62 ± 43.39 ng/mL. These findings indicate that both groups were in a state of heightened inflammatory activity, as reflected by their significantly elevated FC levels, suggesting that both groups experienced considerable intestinal inflammation. The relationship between FC, an inflammatory marker, and PRL is not entirely linear and may be influenced by multiple factors, such as variations in the gut microbiota or different patterns of local immune responses.

We also observed that the second visit data showed a decrease in FC levels for both groups, although the levels remained above the normal range (normal group: 497.92 ± 89.42, abnormal group: 487.60 ± 66.98). This suggests that while both groups experienced a reduction in FC levels, they remained in a state of ongoing inflammation.

The complex relationship between FC and PRL could be reflective of the multifaceted nature of immune regulation in Crohn’s disease. FC levels may not only serve as a marker of gut inflammation but may also interact with systemic immune responses, which in turn affect PRL regulation. It is possible that higher FC levels reflect a specific immune response that helps maintain normal PRL levels during periods of inflammation. Further studies are needed to investigate the co-regulation of FC and PRL in inflammatory conditions and their implications for disease activity and treatment outcomes.

Obesity and malnutrition in CD are also gaining attention (29, 30). This study found that PRL monitoring had little significance in response to nutritional indicators, such as Alb and BMI. Folic acid is a water-soluble vitamin (31); several studies have shown that patients with IBD have symptoms of folic acid deficiency due to restricted intestinal function (32). However, in the present study, folate levels in both the normal and abnormal PRL groups were within the normal range, whereas the significant difference in folate levels between the two groups disappeared following the multivariate analysis. Therefore, the importance of folic acid in assessing whether PRL is abnormal seems to be minimal.

The observed reduction of PRL levels parallel to decreases in CRP following therapy indicates a potential association between PRL secretion and the inflammatory state. PRL receptors are expressed on lymphocytes and macrophages, and PRL has been shown to enhance IL-6 and TNF-*α* production under inflammatory conditions (33–35). It is plausible that elevated PRL levels represent a compensatory immune response that supports mucosal repair during inflammation, or conversely, may perpetuate chronic immune activation in susceptible individuals (36). This dual behavior aligns with the context-dependent immunological role of PRL observed in other autoimmune settings.

Interestingly, in our cohort, patients with persistently high PRL levels after treatment tended to exhibit elevated CRP and hemoglobin values, suggesting that PRL maintenance may be linked to subclinical inflammation or altered iron metabolism. This observation warrants further mechanistic investigation, as PRL has been shown to influence erythropoietin signaling and hematopoietic function (37–39). Moreover, differences between male and female patients highlight the importance of considering sex-specific hormonal regulation when interpreting PRL-related findings (40).

Our study has several limitations. First, as a single-center study from a colorectal surgery department, our cohort had an exceptionally high prevalence of perianal surgery (approximately 95%), indicating enrichment for severe, perianal-dominant CD. This limits the representativeness of our findings for the broader CD population (41). In addition, many patients did not require multiple procedures and chose other medical centers for ongoing treatment, resulting in a high rate of missed visits (46%). As PRL tests are not routinely performed to evaluate patients with CD, the time was not uniform, although we followed up with our patients several times. Therefore, in analyzing the treatment feedback, we used a before and after control analysis rather than observations at several fixed time points and provided a visual natural course, as shown previously (19), which may make the experiment much less preparatory. Third, a significant baseline imbalance in reproductive history existed between groups, with controls having a higher rate of prior childbirth. This is not unexpected, as reduced fertility in CD is multifactorial, relating to disease activity, prior surgeries, medication use, and voluntary childlessness. Although we adjusted for reproductive history in our multivariable models (where it was not an independent predictor of PRL status, see Table 3), its potential residual confounding effect on group-wise PRL comparisons cannot be entirely ruled out. This imbalance underscores that our control group represents a different reproductive population, and thus, our findings regarding absolute PRL levels are most interpretable within the context of the associated clinical and inflammatory factors analyzed, rather than as direct, unadjusted comparisons. Another important limitation is the lack of systematic assessment of endoscopic activity at baseline and during follow-up, which restricts our ability to evaluate objective inflammatory changes and mucosal healing. Fifth, the longitudinal assessment of prolactin was limited to a single follow-up time point within 14–30 weeks after treatment initiation. This design does not capture the dynamic fluctuations of prolactin across the entire disease course, including periods of relapse and sustained remission. Future studies with serial measurements over extended periods are needed to define the longitudinal profile of prolactin in CD. Finally, the 30-week observation period seems too short compared to the cycle of biological therapy in patients with CD, which also leads to an inadequate observation of patients who do not respond to biologicals.

## Conclusion

Our study confirmed the prevalence of abnormally high PRL levels in patients with CD, a phenomenon that correlates with indicators of inflammation. Furthermore, hyperprolactinemia in patients with CD may suggest a better efficacy of biological therapy in reducing intestinal inflammation. PRL testing during CD treatment could provide some indication of the inflammatory prognosis.

In summary, this prospective cohort study confirmed the abnormal elevation of prolactin in patients with Crohn’s disease and revealed its dynamic association with inflammatory markers and treatment outcomes. These findings suggest that prolactin may play a dual immunomodulatory role in Crohn’s disease and serve as a supplementary biomarker reflecting disease activity and therapeutic response. Further large-scale, multi-center, and mechanistic studies are warranted to validate these findings and explore the therapeutic implications of modulating prolactin pathways in inflammatory bowel disease.

## Data Availability

The original contributions presented in the study are included in the article/supplementary material, further inquiries can be directed to the corresponding authors.
